# The prevalence of depression among parents of children/adolescents with type 1 diabetes: A systematic review and meta-analysis

**DOI:** 10.3389/fendo.2023.1095729

**Published:** 2023-03-01

**Authors:** Zhichao Chen, Jing Wang, Ciriaco Carru, Donatella Coradduzza, Zhi Li

**Affiliations:** ^1^ Department of Biomedical Sciences, University of Sassari, Sassari, Italy; ^2^ Department of Cardiology, Second Affiliated Hospital of Shantou University Medical College, Shantou, China; ^3^ Department of Obstetrics and Gynecology, Second Affiliated Hospital of Shantou University Medical College, Shantou, China

**Keywords:** parental depression, parental mental illness, type 1 diabetes mellitus (T1DM), children, adolescents, systematic review and meta-analysis

## Abstract

**Background:**

Emerging research indicates that depression among parents of children/adolescents with type 1 diabetes mellitus (T1DM) has increased significantly. However, the prevalence rates reported by different studies vary substantially.

**Methods:**

Seven databases were systematically searched (Pubmed, Embase, MEDLINE, Scopus, Web of Science, Cochrane Library, PsycInfo) from the inception to 15th October 2022. We pooled prevalence rates from each study with a random-effect model. We conducted a stratified meta-analysis to identify the potential sources of heterogeneity among studies. The GRADE (Grading of Recommendations, Assessment, Development and Evaluations) approach was utilized to evaluate the quality of evidence.

**Results:**

Twenty-two studies were included, with a total of 4639 parents living with type 1 diabetic children. Overall, the pooled prevalence rate of depression or depressive symptoms was 22.4% (95%CI 17.2% to 28.7%; *I* ^2^ = 96.8%). The prevalence was higher among mothers (31.5%) than fathers (16.3%) as well as parents of children (aged < 12 years) with T1DM (32.3%) than those with adolescents (aged ≥ 12 years) (16.0%).

**Conclusion:**

Our research suggests that more than 1 in 5 parents of type 1 diabetic children/adolescents worldwide suffer from depression or depressive symptom. Depression screening and interventions are required for parents of children with T1DM.

**Systematic review registration:**

https://www.crd.york.ac.uk/prospero/, identifier (CRD42022368702).

## Introduction

The prevalence of T1DM is increasing globally, making it the third most prevalent chronic childhood condition (1). A recent study suggests that approximately 1.2 million children and adolescents under the age of 20 have T1DM worldwide (2). T1DM is a complex disease requiring daily insulin injections, glucose monitoring and a strict diet (3). During childhood, parents play a crucial role in managing and monitoring T1DM in their children or adolescents. Type 1 diabetes usually occurs without warning, compelling parents to make multiple life changes in a short period of time. For the parents of those children, stress and numerous life changes can be overwhelming or even worse (4, 5). Indeed, caring for a child with T1DM is a massive challenge for parents due to their children’s cognitive and verbal immaturity (6). Meanwhile, Parents frequently express grave concerns and feelings of guilt regarding hypoglycaemia and other complications. All of these factors contribute to an increased risk of depression and parental stress among parents of type 1 diabetic children/adolescents (7). In a European study of parents coping with children with T1DM, it showed that a significant proportion of parents suffered from post-traumatic stress disorder (22% of fathers and 24% of mothers) (8). However, relatively few studies exist to explore the feelings and mental problems that parents ascribe to those experiences.

Evidence indicates that depression is prevalent among the parents of children with T1DM compared to the parents of healthy children (9–11). The prevalence rates reported by various countries and regions ranged from 5% to 73.5% (12–16). Several factors contribute to the wide variation in depression prevalence, including (a) different assessment tools for evaluating parental depression and depressive symptoms across studies (b) variation in social health care systems in supporting type 1 diabetes and (c) the differences in the characteristics of children, adolescents and their parents included in studies. Consequently, it was improbable that a single study would yield a relatively precise prevalence of depression.

Parental psychological problems are frequently connected with an increased risk of childhood depression, anxiety and internalised problems in the general population (17, 18). In the population of T1DM, parents with psychological problems have negatively impacted their children’s mental health and worsened T1DM outcomes and diabetes management (19, 20). In the meantime, parental psychological problems may also lead to insufficient or excessive involvement in the diabetes care routine, resulting in distress and poor diabetes management in their children (21, 22). Indeed, focusing on the severity of depression in parents is essential for implementing possible early screening and appropriate intervention strategies to improve the management of T1DM in children and adolescents.

Until now, there are no reviews and meta-analysis to analyze systematically and summarise the prevalence of depression among parents of children/adolescents with T1DM. Thus, we conducted this meta-analysis to assess the prevalence of depression among parents of children/adolescents with T1DM. We also aimed to survey whether gender, type of tools for assessment, location, age of children or income level influenced the prevalence rates. Hopefully, it can raise awareness of the problem among society and clinicians, promoting a screening of patients of children with T1DM for the symptoms of depression and other mental problems.

## Materials and methods

This systematic review and meta-analysis followed the protocols from the Preferred Reporting Items for Systematic Reviews and Meta-Analyses (PRISMA) (23) and was registered in the PROSPERO database (number: CRD42022368702). The PRISMA checklist is available in the **Supplementary Material** (see **Supplementary File 1**).

### Literature search

Systematically searches were conducted in the following electronic databases: Pubmed, Embase, MEDLINE, Scopus, Web of Science, Cochrane Library and PsycInfo from the inception to 15th October 2022. The keywords we used for searching were “depression,” “depressive disorder,” “depression symptom(s),” “parent(s),” “parental,” “caregiver(s),” “mother,” “father,” “diabetes mellitus, type 1,” “insulin-dependent diabetes mellitus,” and “type 1 diabetes”. We also manually searched the reference lists for the additional records (see **Supplementary File 2**).

### Inclusion and exclusion criteria

Included studies satisfied the following criteria:

1) Participants included parents of children/adolescents with type 1 diabetes;
2) The prevalence rates of depression among parents or the data for calculating were provided;
3) The tools for assessing depression and diagnostic criteria were clarified in the articles;
4) Study population ≥ 50 participants to reduce bias in results due to small sample sizes;
5) Participants’ children were younger than 18 years old;
6) Studies were published in English.

Studies were excluded if they 1) were reviews, commentaries, letters to the editor, conference papers and books; 2) included participants with cognitive impairment and inability to complete a depression assessment.

### Study selection

**Figure 1:**
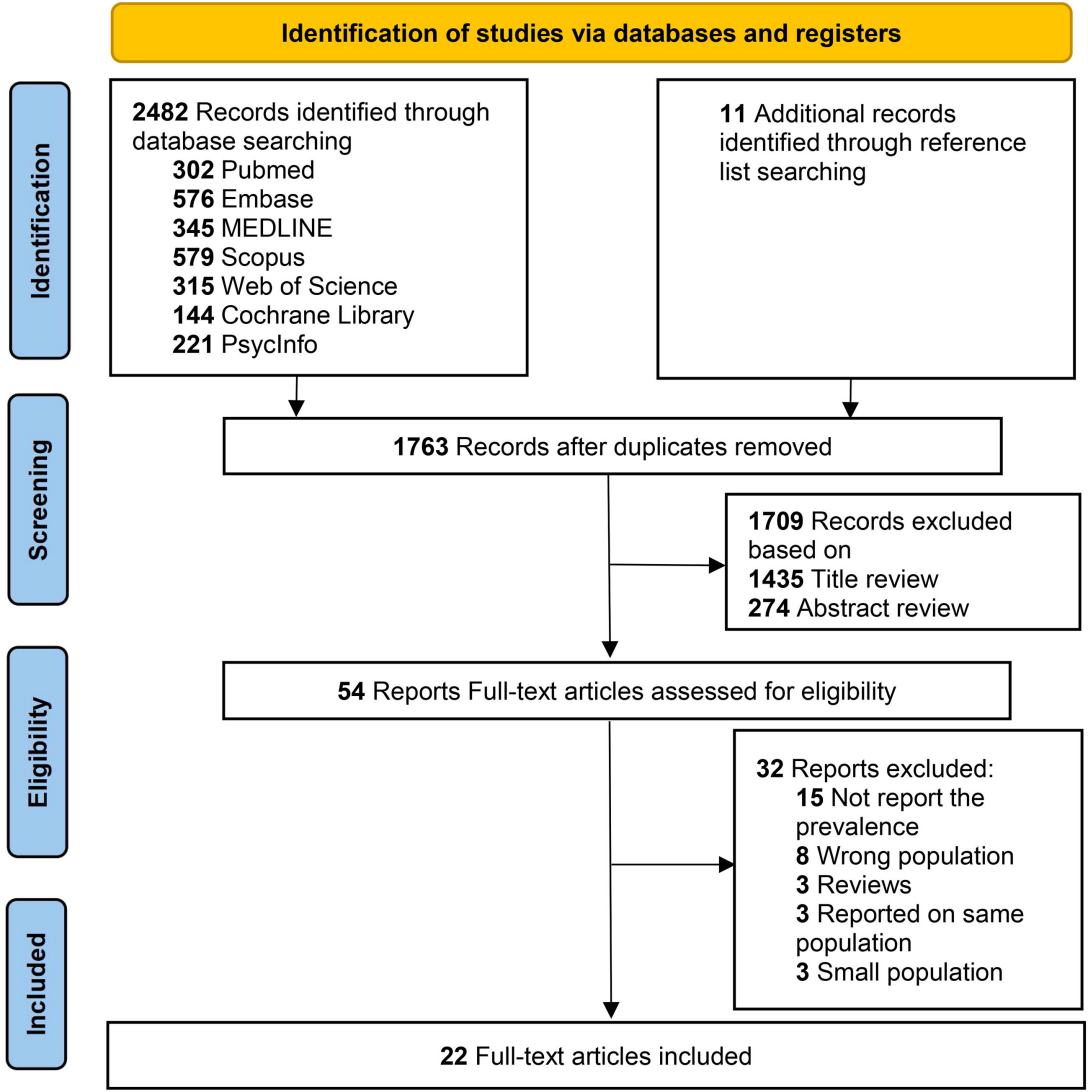
PRISMA flow diagram.

Two investigators (ZC and JW) independently searched the literature and reviewed study titles and abstracts. Studies that met the inclusion criteria were assessed for full-text review. Two investigators were responsible for the detailed analysis, quality assessment and data extraction of the included studies. A third independent investigator (CC) was consulted in the event of any disagreement between the investigator. **Figure 1** illustrates a summary of the study selection procedure.

### Data extraction

Two independent investigators (ZC, JW) extracted the following data from each study included: author, year of publication, country, depression assessment tools for parents, age of participants, age of children/adolescents, race, sample population, positive cases of depression and the corresponding prevalence rate. In addition, we analyzed corresponding data from mothers and fathers as well as from children and adolescents.

### Risk of bias

We evaluated the quality of each study by using the Joanna Briggs Institute (JBI) critical appraisal tool and a nine-items questionnaire for prevalence studies (24). The score of each study was calculated according to JBI checklist by selecting “yes”, “no”, “not clear” and “not applicable” for each item. The total quality score ranges between 0 and 9.

### Statistical analysis

We used the Comprehensive Meta-analysis Software (version 3.0) to calculate pooled prevalence rates and 95% confidence intervals (95% CI) of participants living with children/adolescents with T1DM. *P* values < 0.05 was considered statistically significant in all analyses. We used a random-effects model to pool the prevalence rates from each study due to variability across studies. Pooled prevalence is presented as an event rate (i.e., 0.20) in figures, whereas it is reported as prevalence (i.e., 20.0%) in tables and text. The heterogeneity of prevalence rates across studies was evaluated using the Q test, and the values of *I* ^2^ statistics, such as 25%, 50% and 75%, were regarded as the cutoffs for low, medium and high heterogeneity, respectively (25). Stratified meta-analysis (gender, tools for depression assessment, quality score, regions, age of parents, age of T1DM children) were performed to investigate the potential source of heterogeneity across studies. One-way ANOVA was used to detect variation between different subgroups. Sensitivity analysis was conducted to determine the influence of individual studies on the pooled prevalence by sequentially removing one study at each time (26). Potential publication bias was examined by funnel plots and Egger’s test (27).

### Level of evidence

The quality of evidence for prevalence rate was categorized into the following level: high quality, moderate quality, low quality and very low quality by using GRADE approaches, which includes five factors that can degrade the quality of evidence: Risk of bias, indirectness, inconsistency, imprecision, publication bias (28). We used the GRADEpro software to perform the quality assessment.

## Results

### Study selection

As shown in the flow diagram from PRISMA (**Figure 1**), 2482 articles were identified according to our search strategy from electronic databases. 11 more records were manually acquired from the reference lists. 54 studies were relevant for a full-text review and 32 studies were excluded due to wrong population, small population, type of research, duplicate population data and lack of data on prevalence. Finally, a total of 22 non-duplicated studies met the inclusion criteria (12–15, 19, 29–44).

### Characteristics of included studies

**Table 1** demonstrates the characteristics of the 22 included studies. All studies included were published from 1985 to 2022, with a total of 1389 cases of depression and 4639 participants. The sample sizes below 50 were excluded and ranged from 60 to 1079 participants. 13 studies (59.1%) were from North America, 5 studies (22.7%) were from Europe, 2 studies were from Asia (9.1%) and 2 studies were from South America (9.1%). 7 studies (31.8%) reported the prevalence rates of both genders of parents. Seven instruments were used across studies for depression assessment, including the Center for Epidemiologic Studies Depression Scale (CES-D), Beck depression inventory (BDI-II), Patient health questionnaire (PHQ-9), Hospital Anxiety and Depression Scale (HADS), Brief symptom inventory 18 (BSI 18), EuroQol-5D (EQ-5D), World Health Organization-five well-being index (WHO-5).

**Table 1:** Selected Characteristics of 22 studies included in meta-analysis

Source	Country	Publication	Size,	Prevalence (%),	Fathers, No. (n)	Mothers, No. (n)	Age,	Age,	Tool,
		Year	(n)	cases (n)	Prevalence (%)	Prevalence (%)	Parents	Children	Cutoff
Kovacs et al	US	1985	107	29.0%, 31	38, 13.2%	69, 37.7%	39.6 (6.8)	11.0 (1.5)	BDI-II 10
Horsch et al	UK	2007	60	16.7%, 10	NA	60, 16.7%	40.2 (5.9)	10.3 (4.1)	HADS 8
de Wit et al	Netherlands	2007	91	14.3%, 13	NA	NA	NA	14.9 (1.1)	CES-D 16
Jaser et al	US	2008	108	22.2%, 24	NA	108, 22.2%	39.8 (5.5)	9.9 (1.5)	CES-D 16
Streisand et al	US	2008	102	73.5%, 75	NA	NA	40.2 (7.2)	9.7 (4.0)	CES-D 16
Williams et al	US	2009	180	23.3%, 42	NA	NA	NA	14.4 (2.4)	CES-D 16
Eckshtain et al	US	2009	100	10%, 10	NA	NA	NA	14.4 (2.2)	BSI-18 65
Grey et al	US	2009	67	23.9%, 16	NA	67, 23.9%	37.2 (5.6)	4.77 (1.5)	CES-D 16
Driscoll et al	US	2010	108	33.3%, 36	18, 16.7%	90, 36.7%	37.6 (8.4)	8.1 (2.5)	CES-D 16
Hansen et al	US	2012	125	23.2%, 29	43, 18.6%	82, 25.6%	42.3 (5.7)	10.8 (1.6)	HADS 8
Malerbi et al	Brazil	2012	1079	51.2%, 547	115, 32.2%	964, 52.9%	38.6 (7.6)	11.4 (3.9)	EQ-5D NA
Moreira et al	Portugal	2013	104	10.6%, 11	NA	NA	42.0 (6.0)	12.4 (3.7)	HADS 11
Mackey et al	US	2014	225	20.9%, 47	NA	225, 20.9%	NA	12.7 (1.2)	BDI-II 14
Jaser et al	US	2014	118	18.0%, 21	NA	118, 18.0%	44.2 (5.8)	12.8 (2.0)	CES-D 16
Capistrant et al	India	2018	165	9.7%, 16	82, 6.1%	83, 13.3%	41.3 (6.5)	13.1 (3.2)	PHQ-9 10
McConville et al	US	2020	128	29.7%, 38	16, 12.5%	112, 26.8%	36.6 (6.4)	7.5 (1.30	CES-D 16
von Borries et al	Chile	2020	86	25.6%, 22	NA	86, 25.6%	43.0 (8.1)	14.0 (2.3)	BDI-II 14
Noser et al	US	2020	126	25.4%, 32	14, 14.3%	112, 26.8%	36.6 (6.4)	7.5 (1.3)	CES-D 16
Nguyen et al	Netherlands	2021	121	5%, 6	NA	NA	46.0 (4.6)	15.0 (1.9)	PHQ-9 10
Inverso et al	US	2022	157	26.8%, 42	NA	NA	34.1 (7.0)	4.5 (1.7)	CES-D 16
Stahl−Pehe et al	German	2022	1058	27.6%, 292	NA	NA	NA	14.3 (1.5)	WHO-5 50
Luo et al	China	2022	224	12.9%, 29	NA	NA	39.9 (5.0)	13.5 (2.5)	PHQ-9 10

BDI-II Beck depression inventory; HADS Hospital Anxiety and Depression Scale; CES-D Center for Epidemiologic Studies Depression Scale; BSI-18 Brief symptom inventory 18; EQ-5D EuroQol-5D; PHQ-9 Patient health questionnaire; WHO-5 World Health Organization-five well-being index; NA Not applicable.

### Risk of bias

**Table 2** shows the risk of bias and the quality assessment of the included studies in our meta-analysis using the JBI checklist. 17 studies (77.3%) were missing sample size estimates and adequate sample sizes and 2 studies (9.1%) did not report the source of participants. The vast majority of the included studies (18/22) described the sources and characteristics of participants in detail. All studies (100%) used standard and valid instruments for assessing depression. Overall speaking, the quality of the studies was fair (6–7) to high (8–9).

**Table 2:** Qualities of studies included in meta-analysis.

Study name	Response									
	Q1	Q2	Q3	Q4	Q5	Q6	Q7	Q8	Q9	Score
Kovacs et al	N	Y	N	Y	Y	Y	Y	Y	Y	7
Horsch et al	Y	U	N	Y	Y	Y	Y	Y	U	6
de Wit et al	Y	Y	N	N	Y	Y	Y	Y	Y	7
Jaser et al	Y	Y	N	Y	Y	Y	Y	N	Y	7
Streisand et al	Y	Y	U	Y	Y	Y	Y	Y	Y	8
Williams et al	Y	Y	U	N	Y	Y	Y	U	Y	6
Eckshtain et al	Y	Y	N	N	Y	Y	Y	U	Y	6
Grey et al	Y	Y	N	Y	Y	Y	Y	Y	Y	8
Driscoll et al	Y	Y	N	Y	Y	Y	Y	Y	Y	8
Hansen et al	Y	Y	N	Y	Y	Y	Y	Y	U	7
Malerbi et al	Y	Y	Y	Y	Y	Y	Y	Y	Y	9
Moreira et al	Y	Y	N	Y	Y	Y	Y	Y	U	7
Mackey et al	Y	Y	Y	Y	Y	Y	Y	Y	Y	9
Jaser et al	Y	Y	U	Y	Y	Y	Y	Y	Y	8
Capistrant et al	N	Y	U	Y	Y	Y	Y	Y	Y	7
McConville et al	Y	Y	N	Y	Y	Y	Y	Y	Y	9
von Borries et al	Y	Y	N	Y	Y	Y	Y	Y	U	7
Noser et al	Y	Y	N	Y	Y	Y	Y	Y	Y	8
Nguyen et al	Y	Y	N	Y	Y	Y	Y	Y	U	6
Inverso et al	Y	Y	Y	Y	Y	Y	Y	Y	U	8
Stahl−Pehe et al	Y	Y	Y	N	Y	Y	Y	Y	Y	8
Luo et al	Y	Y	Y	Y	Y	Y	Y	Y	Y	9

Q1-Q9: Question 1 to question 9 used to assessment the quality of studies reporting prevalence data in the JBI critical appraisal checklist.

Q1. Was the sample frame appropriate to address the target population? Q2. Were study participants sampled in an appropriate way?

Q3. Was the sample size adequate? Q4. Were the study subjects and the setting described in detail?

Q5. Was the data analysis conducted with sufficient coverage of the identified sample?

Q6. Were valid methods used for the identification of the condition?

Q7. Was the condition measured in a standard, reliable way for all participants? Q8. Was there appropriate statistical analysis?

Q9. Was the response rate adequate, and if not, was the low response rate managed appropriately?

Y, yes; N, no; U, unclear; NA, not applicable.

### Pooled prevalence of depression among parents of children/adolescent with T1DM

**Figure 2:**
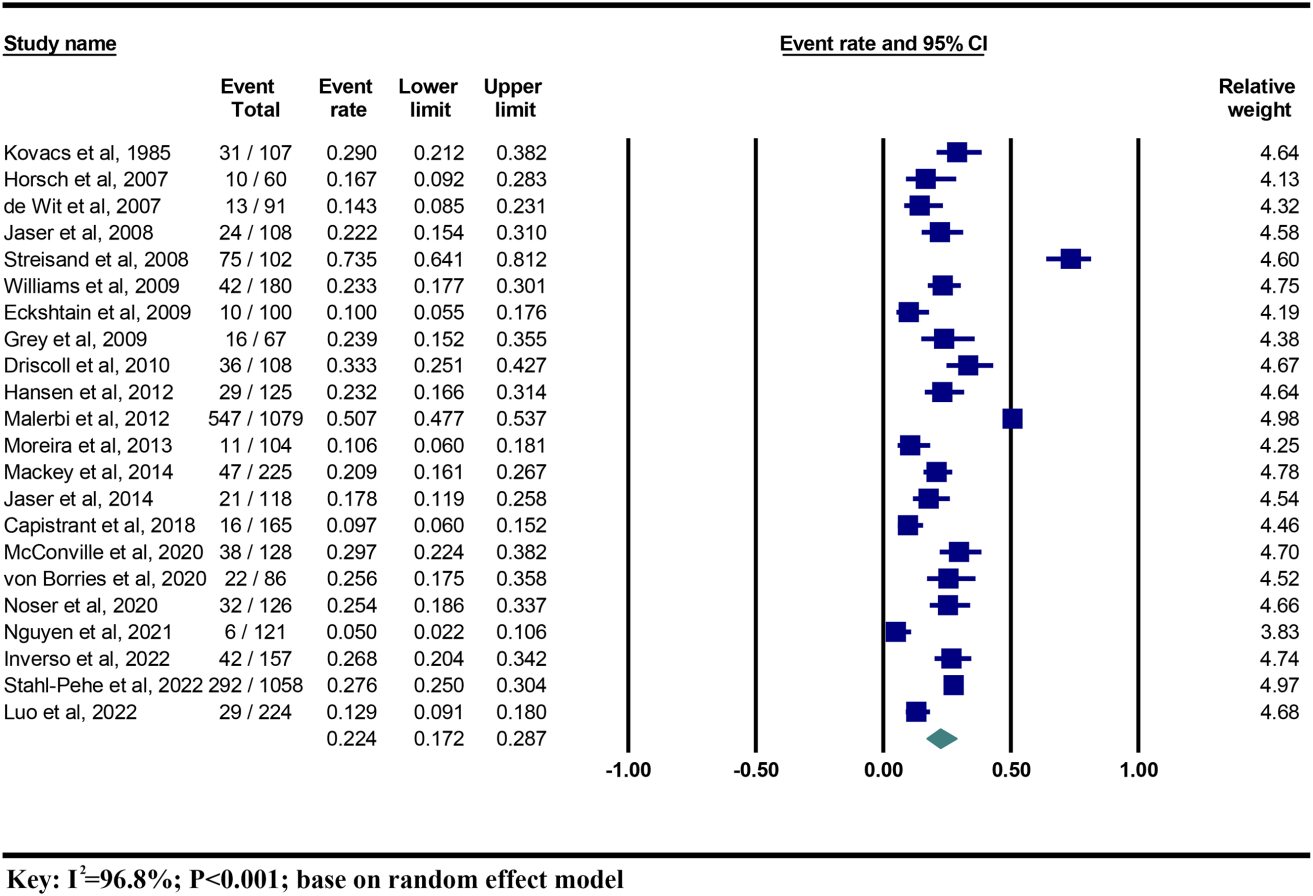
The prevalence of parental depression among children/adolescents with T1DM.

**Figure 3:**
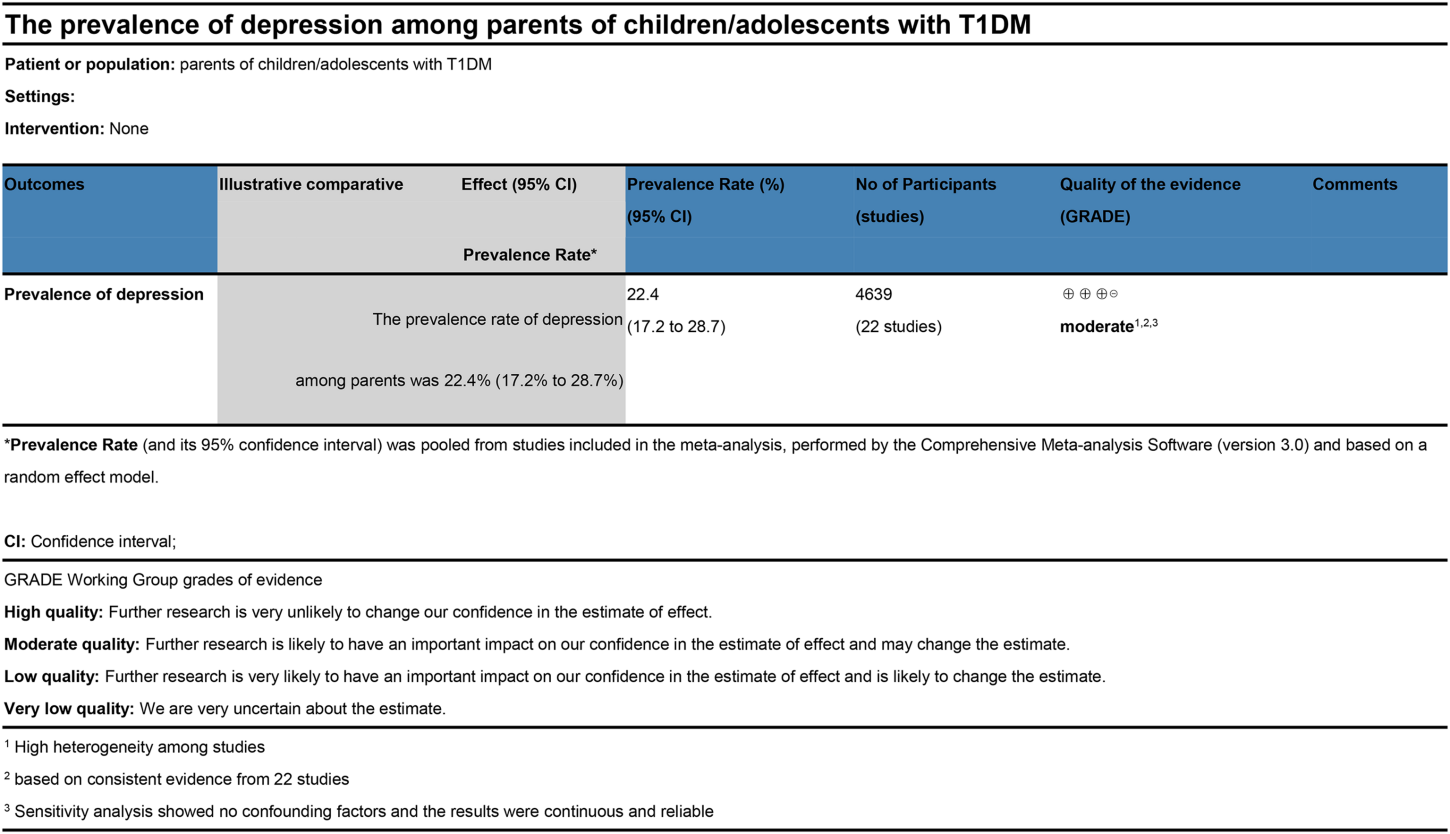
Level of evidence (GRADE).

The pooled prevalence of depression among parents of children/adolescents with T1DM was 0.224 (95%CI 0.172 to 0.287; *I* ^2 =^ 96.8%) or 22.4% using a random-effect model (**Figure 2**). The quality assessment of GRADE approaches reported that the level of evidence was moderate (**Figure 3**).

### Subgroup analysis

Stratified meta-analysis was conducted by the following categories: gender of parents (father or mother), tools for assessment (CES-D, BDI-II, PHQ-9, HADS, others), quality score (fair or high), location, age groups of parents (≥40 years or <40 years), age groups of children (≥12 years or <12 years).

As shown in **Table 3**, Mothers were associated with significantly higher prevalence rates of depression than fathers (31.5% *vs* 16.3%, F=7.3, *P*=0.019). We found the prevalence rates increased in studies that used other instruments (BSI 18, EQ-5Q, WHO-5) (29.6%), followed by CES-D (28.9%) and BDI-II (24.1%), although heterogeneity between groups was not statistically significant (F= 1.43, *P*=0.268).

**Table 3:** Stratified prevalence of depression among parents of chlidren/adolescent with type 1 diabetes.

Subgroups	Study, n	Prevalence	Heterogeneity among the studies		Heterogeneity between	
		(95%CI)	*I^2^%*	*P* value	groups (*F* and *P* value)	
Gender					7.3	0.019
Mother	7	31.5 (17.9-45.0)	95.9	<0.001		
Father	7	16.3 (7.5-25.1)	77.9	<0.001		
Tools for assessment					1.43	0.268
CES-D	10	28.9 (19.5-38.4)	93.5	<0.001		
BDI-II	3	24.1 (19.2-29.0)	25.3	0.262		
PHQ-9	3	9.1 (4.4-13.8)	72.8	0.025		
HADS	3	16.6 (8.6-24.6)	70.9	0.032		
Others	3	29.6 (9.1-50.1)	99.0	<0.001		
Quality Score					6.02	0.023
6-7	11	16.7 (11.8-21.6)	84.5	<0.001		
8-9	11	24.1 (17.4-30.8)	97.3	<0.001		
Location					2.39	0.102
North America	13	27.5 (20.0-34.9)	92.6	<0.001		
South America	2	38.5 (13.9-63.1)	96.1	<0.001		
Asia	2	11.4 (8.2-14.5)	2.1	0.312		
Europe	5	14.8 (3.9-25.8)	96.0	<0.001		
Age group of parents					0.46	0.508
≥40 years old	8	22.5 (10.0-35.0)	96.9	<0.001		
<40 years old	9	28.3 (17.0-39.5)	96.6	<0.001		
Age group of children					8.92	0.007
≥12 years old	11	16.0 (10.6-21.5)	92.3	<0.001		
<12 years old	11	32.3 (22.3-42.3)	95.5	<0.001		
Annual Family Income					0.40	0.543
> $30,000	6	29.6 (12.4-46.7)	96.7	<0.001		
< $30,000	5	21.9 (1.8-42.0)	98.9	<0.001		
Average Education Level					4.07	0.090
≤ High school	4	13.5 (8.5-18.6)	59.1	0.062		
≥ College	4	24.2 (18.0-30.3)	68.9	0.022		

The prevalence estimated from studies limited to South America (38.5%) and North America (27.5%) was higher than those limited to Europe (14.8%) and Asia (11.4%). However, the difference between groups of location was not statistically significant (F= 2.39, *P*=0.102).

Concerning the quality of studies, an increased prevalence was seen among studies with better quality scores (high quality 24.1% *vs* fair quality 16.7%, F= 6.02, *P*=0.023). Moreover, we found that the prevalence was higher among parents of children (aged < 12 years) than those living with adolescents (aged ≥ 12 years) (32.3% *vs* 16.0%, F= 8.92, *P*=0.007).

The prevalence were higher as the annual household income increased (29.6% *vs* 21.9%, F= 0.40, *P*=0.543) and as those parents with better education background (24.2% *vs* 13.5%, F= 4.07, *P*=0.090), which were not statistically significant.

### Sensitivity analysis and publication bias

**Figure 4:**
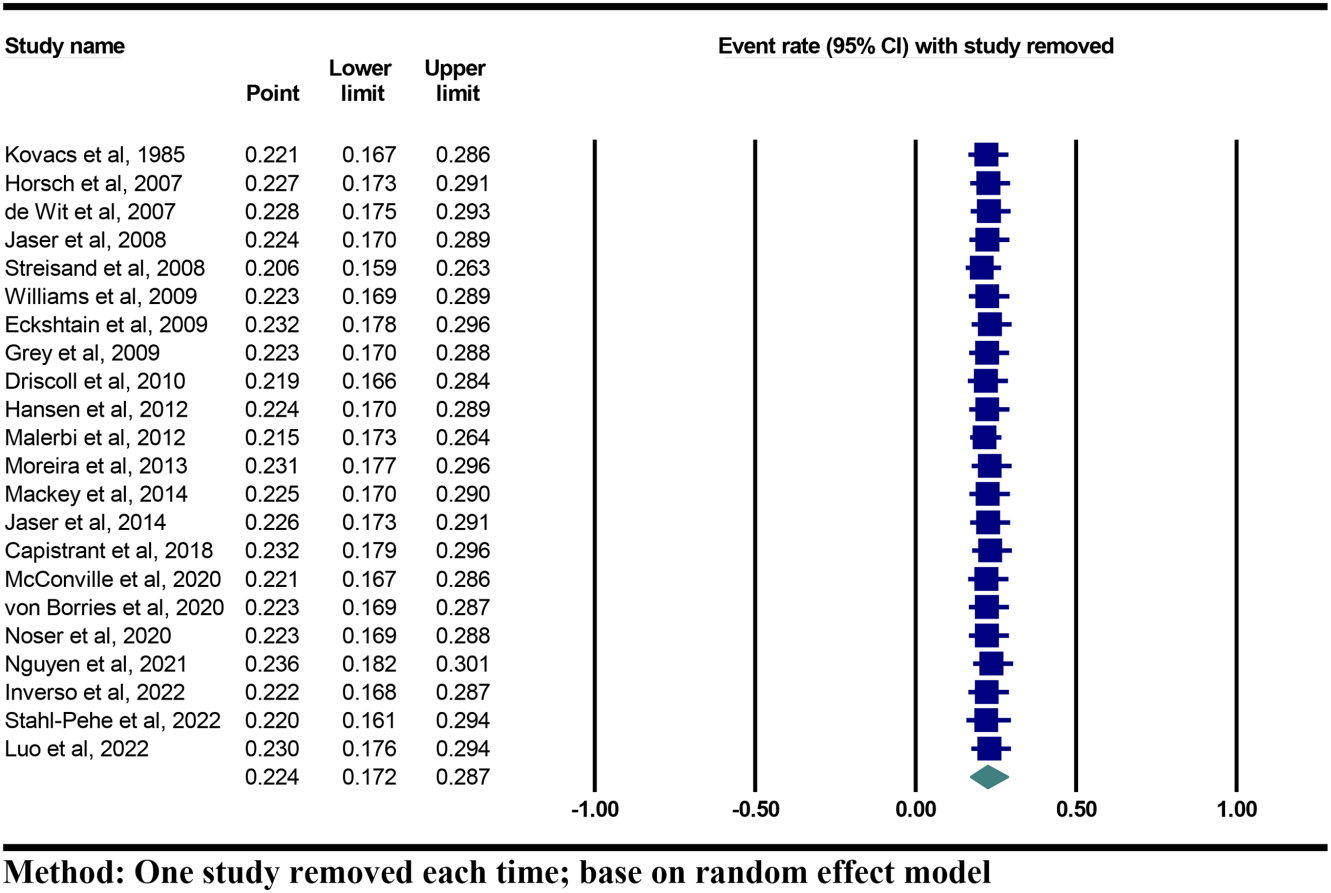
Forest plots of leave-one-out sensitivity analysis: based on random effect model.

Sensitivity analysis was conducted using an omitting-one-out analysis to estimate the possible source of heterogeneity across studies included in our study. This approach suggested that the pooled prevalence rates of depression among parents remained stable and consistent. The pooled prevalence rates of depression among parents vary from 20.6% (95%CI 15.9% to 26.3) to 23.6% (95%CI 18.2% to 30.1%) (**Figure 4**).

There was a significant asymmetry in the funnel plot, representing the presence of publication bias by visual examination (**Figure S1**). However, there was no potential publication bias detected with the result of Egger’s test (B = -1.97, SE = 3.10, *P* = 0.534).

## Discussion

### Main findings

This study, to our knowledge, is the first systematic review and meta-analysis on the prevalence of depression among parents of type 1 diabetic children/adolescents. Our review has assembled data from 22 studies involving 4639 subjects in ten countries from four continents. Meanwhile, we reported the prevalence of depression among parent males and females and parents caring for children and adolescents. At the same time, we found that the variability between the finding across different studies was strongly associated with three factors: the gender of parents, the quality of the studies judged by the JBI checklist and the age of the participants’ children.

### Prevalence of depression

Overall, the present analysis demonstrated that a surprisingly high proportion of parents caring for their children with T1DM had depression disorder or depressive symptoms (22.4%). The summary prevalence rates (31.5% in mothers *vs* 16.3% in fathers) suggested that depression affected every family member with significant variability. In addition, a comparison of the prevalence of depression among parents with T1DM children (aged < 12 years) and parents with T1DM adolescents (aged ≥ 12 years) suggested that the incidence of depression in parents is likely to double when raising younger children (32.3% *vs* 16.0%).

### Comparison, explanation and connection

Depression is a significant cause of disability worldwide and it is estimated that roughly 5% of adults are suffering from depression according to an extensive global survey in 2022 (45). The prevalence of depression among parents of children/adolescents with T1DM in our study (22.4%) is 4.48 times more prevalent than the general population among adults. There are a couple of reasonable explanations for the higher prevalence of depression among parents of children with paediatric diabetes compared to the general population. One of the potential reasons for the question would be the substantial emotional devastation in parents according to the announcement of the diagnosis of their children with diabetes. Over 1 in 3 parents reported distress at the diagnosis and the effect would last for years (20, 46). The other possible reason is the overwhelming sense of responsibility in managing children’s blood glucose levels even though they had mastered executing a complex and demanding daily diabetes treatment for their children. High-stress levels associated with childhood diabetes have been described as risk factors for depression (47). Finally, children/adolescents with type 1 diabetes had great opportunities to experience social discrimination, marginalization and stigma as well as their parents when compared to the healthy population (48, 49).

As we expected, our review showed that a higher proportion of mothers experienced depressive symptoms or depressive feelings than fathers. Indeed, this was because mothers were the prime caregivers in most families, took on a higher task of diabetes management and paid more attention to the inner pain of children with T1DM (50, 51). Furthermore, our meta-analysis also showed that the prevalence of depression increased in parents caring for younger children than those living with older adolescents of T1DM. The possible reason for the differences was higher levels of paediatric parenting stress which was related to more significant parental depressive symptoms when raising a younger child with type 1 diabetes (41). In addition, it is essential to notice that other moderators (i.e., socio-economic status, regions and age of the parents) might potentially impact the prevalence of depression among parents of children with T1DM.

In terms of practice, parental depressive symptoms and diabetes-related stress would contribute to the weakening of parental functioning, impacting the management effectiveness of diabetes. For instance, several studies reported that parental emotional problems were directly and indirectly associated with a level of HbA1c, one of the leading indicators for diabetes control (34, 52). Evidence indicated that children of parents with depression were at higher risk of developing mental disorders, including depression, in childhood and adolescence (53). Thus, parental depression issues should not be treated as an isolated problem but rather an essential part of managing type 1 diabetes.

### Strength and limitations

Our systematic review and meta-analysis has a couple of strength: 1. It was the first study to quantify and summarise the prevalence of depression among parents of children with type 1diabetes using scientific and statistical methods. 2. We conducted subgroup analyses of multiple factors to show differences between the prevalence of parental depression by gender, by the age of children, by region, and by assessment tool. 3. We applied the JBI critical appraisal tool to assess the risk bias of each included study and the GRADE approaches to evaluate the quality of evidence.

We have to admit there are several limitations in our meta-analysis. First, although we analyzed data from 10 countries worldwide, it will become more comprehensive to extract additional data from more countries and regions. Second, all studies included in this meta-analysis used self-report questionnaires to assess the prevalence of depression instead of diagnostic interviews. Third, since we included studies conducted in English, potential data from studies published in other languages could not be extracted.

### The impacts of the findings

Our systematic review and meta-analysis has the following clinical impacts: 1) Further studies are required to investigate the possible sources for the high prevalence of depression among parents of type 1 diabetic children compared to the general population. 2) Public health and medical resources should be coordinated for early diagnosis and intervention of depression among that kind of parents. 3) Since numerous studies have confirmed the association of parental depression with poor diabetes management in children, parental mental health issues should be considered an essential component of paediatric diabetes.

## Conclusions

Our study indicated that the prevalence of depression is distinctly higher among parents of children with T1DM compared to the general population. Encouraging clinicians, schools and work units to detect, interfere and focus on the mental disorders of those parents of children with T1DM might improve the results, leading to a healthier family environment and better management of type 1 diabetes. Moreover, future research is needed to investigate the causes and mechanisms of depression in parents of children with paediatric diabetes.

## Data availability statement

The original contributions presented in the study are included in the article/**Supplementary Material**. Further inquiries can be directed to the corresponding author.

## Author contributions

ZC and JW contributed equally to the study. ZC and JW had the original idea for the study. ZC and JW searched databases and performed the selection of studies. ZC and JW did the statistical analyses and wrote the article’s first draft. CC, DC and ZL contributed to writing and critically editing the manuscript and approved the last version. ZL was the supervisor of this review. All authors contributed to the article and approved the submitted version.

## Supplementary material

Supplementary Figure 1 Funnel plot of publication bias for the prevalence of parental depression among children/adolescents with T1DM.
