# Supplementary figures and images for: The prevalence of depression among parents of children/adolescents with type 1 diabetes: A systematic review and meta-analysis
Source: Front Endocrinol (Lausanne). 2023 Mar 1;14:1095729. doi: 10.3389/fendo.2023.1095729 (PMC10014558; doi:10.3389/fendo.2023.1095729)

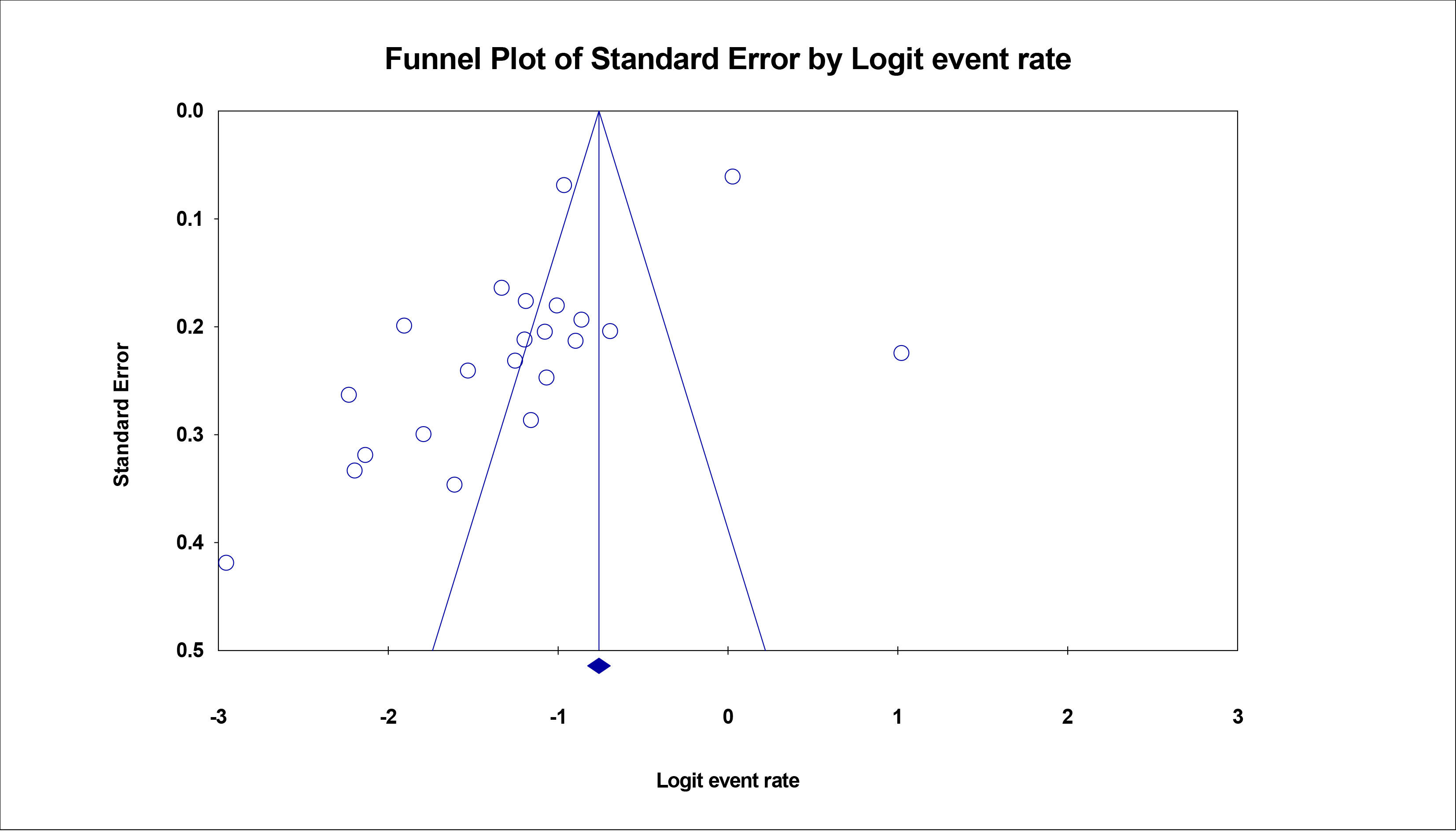

Supplement: Supplementary Figure 1 — Funnel plot of publication bias for the prevalence of parental depression among children/adolescents with T1DM. [file Image_1.tif]
